# Exploring the knowledge, attitude and practice towards disaster medicine preparedness and readiness: A prescriptive insight by the community pharmacists in the United Arab Emirates

**DOI:** 10.1371/journal.pone.0273209

**Published:** 2022-08-25

**Authors:** Ammar Abdulrahman Jairoun, Sabaa Saleh Al-Hemyari, Moyad Shahwan, Nsser M. Alorfi, Faris El-Dahiyat, Md. Sanower Hossain, Miamona Jairoun, Ammar Ali Saleh Jaber

**Affiliations:** 1 School of Pharmaceutical Sciences, Universiti Sains Malaysia, Pulau Pinang, Gelugor, Malaysia; 2 Health and Safety Department, Dubai Municipality, Dubai, United Arab Emirates; 3 Pharmacy Department, Emirates Health Services, Dubai, United Arab Emirates; 4 College of Pharmacy and Health Sciences, Ajman University, Ajman, United Arab Emirates; 5 Center of Medical and Bio-allied Health Sciences Research, Ajman University Ajman, Ajman, United Arab Emirates; 6 Department of Pharmacology and Toxicology, College of Pharmacy, Umm Al-Qura University, Makkah, Saudi Arabia; 7 Clinical Pharmacy Program, College of Pharmacy, Al Ain University, Al Ain, United Arab Emirates; 8 AAU Health and Biomedical Research Center, Al Ain University, Abu Dhabi, United Arab Emirates; 9 Department of Biomedical Science, Kulliyyah of Allied Health Sciences, International Islamic University Malaysia, Kuantan, Malaysia; 10 Faculty of Science, Sristy College of Tangail, Tangail, Bangladesh; 11 Department of Clinical Pharmacy & Pharmacotherapeutics, Dubai Pharmacy College for Girls, Dubai, United Arab Emirates; Quaid-i-Azam University, PAKISTAN

## Abstract

**Background:**

Proper disaster preparedness by community pharmacists has the potential to counter many of the factors that cause threats and high-risk outcomes. Their preparedness and awareness may also help health practitioners and governments to improve disaster response planning.

**Objectives:**

This aims to explore the knowledge, attitude, and practice (KAP) towards disaster medicine preparedness and readiness among community pharmacists in the United Arab Emirates (UAE).

**Method:**

A cross-sectional study was conducted over the ten months among licensed community pharmacists who had three months’ professional experience or more. Face-to-face interviews were carried out and a structured questionnaire was used for data collection. Logistic regression models were used to determine the factors influencing aboucine preparedness and readiness. SPSS Version 24 was used to analyze the data collected.

**Results:**

A total of 500 community pharmacists participated in the study. The average knowledge score was 25.6% with a 95% confidence interval (CI) of [21.7%, 29.4%]. Better knowledge scores were observed in the male gender (OR 2.43; 95% CI 1.05–3.72), participants aged ≥ 31 years old (OR 2.97; 95% CI 1.16–7.6), postgraduates (OR 4.36; 95% CI 2.6–7.3), participants from independent Pharmacies (OR 6.5; 95% CI 4.04–10.4 3), chief pharmacists (OR 3.1; 95% CI 1.86–5.07), participants with 16 years and more experience years (OR 2.42; 95% CI 1.063–5.522) and participants who graduated from regional/international universities (OR 5.92; 95% CI 2.65–13.2). Better attitude and practice about disaster medicine preparedness were observed in postgraduates (OR 2.54; 95% CI 1.26–pharmacists from independent pharmacies (OR 1.35; 95% CI 2.43–2,.66), and chief pharmacists (OR 1.26; 95% CI 1.17–1.35).

**Conclusions:**

It’s essential to provide a continuing education program using different educational strategies urgently needed to improve community pharmacy competencies (e.g. knowledge attitudes, and perceptions) to improve the skills and practices regarding disaster medicine preparedness and readiness.

## Introduction

In recent years, there has been an increasing interest in disaster management, Particularly, Disaster medicine preparedness, which is increasingly recognized as a serious, worldwide public health concern. As disasters have become more prevalent and larger, low- and middle-income countries (LMICs) tend to be more affected. Generally, a disaster is defined as significant damage, loss, or destruction of the functioning of a society, economy, or an environment at any scale due to risky events interacting with conditions of exposure, vulnerability, and capacity, leading to serious physical, mental, environmental and economic impacts [1].

Several researchers have reported different aspects and approaches that are considered to classify disaster management, such as The Four-Step Disaster Management Cycle. These phases include prevention, preparedness, response, and recovery [2, 3]. Of them, preparedness, which is defined as a set of measures undertaken to identify the personnel, training, and equipment needed for different risky events, is essential in disaster management. The duration of disasters is unexpected, and they may last for a short or long period. Then, proper preparedness and readiness are extremely important, especially in the medical field. Disaster medicine preparedness is vital for governments, international organizations health establishments, and medical staff [4, 5]. Major accidents and climate disasters particularly present major challenges to the medical team who are responsible for managing the health consequences. Its occurrence disrupts the normal health conditions among populations. Disaster medicine is a discipline resulting from merging emergency medicine and disaster management [6]. According to the world health organization (2021), more than 2.6 billion people have been affected by natural disasters in the last decade. The frequency and magnitude of different kinds of disasters have increased dramatically over the last decade. Several factors tend to produce disasters in the world, such as earthquakes, hurricanes, tropical storms, floods, wildfires, technological accidents, population growth, man‐made disasters, and environmental degradation [7–9]. However, People exposed to natural hazards in the poorest nations are more than seven times as likely to die than equivalent populations in the richest nations [10].

The United Arab Emirates is located in the Arabian Gulf, subject to many natural disasters due to possible earthquakes, floods, and other natural disasters. Examples of large-scale natural disasters in the Arabian Gulf in the recent two years include the 2020 earthquake in Fujairah, UAE: and the 2021 Hormozgan Province, Iran earthquake. The country has potential exposure to other kinds of disasters including floods and storms. In the last decade, the UAE has experienced several natural disasters such as Cyclone Gonu in 2007 and tropical cyclone Shaheen in 2021. The disasters are managed via The National Emergency Crisis and Disasters Management Authority (NCEMA)which works under National Supreme Security Council. Therefore, UAE has updated many of its guidelines and regulations related to emergencies accordingly. Though, disaster medicine preparedness and readiness and all other disaster regulations are not extensively studied due to the country’s recent legal establishment as a united country [11].

Typically, pharmacists are called upon to fulfill roles related to disasters and are expected to extend their daily duties. The exact pharmacist’s roles in disaster management are well explained in a Delphi study [12], which identified the major roles of pharmacists in Hurricane Katrina such as ensuring that services are provided during and after a disaster. In addition to vaccinations and basic medical checks, other duties include mixing intravenous medications and treating wound infections [12, 13].

In the current pandemic (COVID-19). In addition to providing non-routinely stocked drugs, pharmacists also provide drug information, medication consultation, and Chinese medicine prevention supplements to patients with complex illnesses [14].

Community pharmacists play a crucial role in the medical team. The community pharmacy profession has multiple roles in helping patients and the public, assessing their conditions, dispensing, and optimizing medication use. Pharmacists and other health professionals are required to keep themselves updated about all medical-related knowledge and practical skills. There are different ways of keeping up to date, such as enrolling in traditional or online degree programs, attending online webinars and teleconferences, attending regional and international scientific conferences, joining pharmacists’ associations, and notably researching and reading [15, 16]. Proper disaster preparedness by community pharmacists has the potential to counter many of the factors that cause threats and high-risk outcomes. Their preparedness and awareness may also help health practitioners and governments to improve disaster response planning. However, there are no previous assessments of the knowledge, attitude, and practice toward disaster medicine preparedness and readiness among community pharmacists in the UAE. Therefore, following the importance of this issue, this study aims to explore the knowledge, attitude, and practice t disaster medicine preparedness and readiness among community pharmacists in the United Arab Emirates.

## Materials and methods

### Study design and setting

Using a cross-sectional study, this study aimed to evaluate the knowledge, attitude, and practice of disaster medicine preparedness and readiness among community pharmacists in the UAE. Over the ten months between January 2021 and October 2021, four trained final-year pharmacy students visited community pharmacies in Abu Dubai, Dubai, and the Northern Emirates. During the pilot survey, all the interviewers were trained properly on the questionnaire and the scientific terminology included within the survey. This training program improved the surveyors’ skills and minimized the errors related to the survey.

### Target population

The study subjects were chosen based on the following inclusion and exclusion criteria. Inclusion criteria: community pharmacists who had three months’ professional experience or more and were registered with one of three regulatory bodies (Ministry of Health, Health Authority Abu Dhabi (HAAD), or Dubai Health Authority). Exclusion criteria: pharmacists who were not registered with the above regulatory bodies or who had less than three months’ experience (i.e., recently joined or still serving their probation period).

### Pilot testing

A pilot study was then carried out to test the face validity with 50 community pharmacists whose data were excluded from the final analysis. The pilot study began on January 10, 2021. completed the questionnaire satisfactorily. The outcomes of the pilot study outcomes were employed to calculate the sample size needed for the main research and to check the reliability of the test. As of January 22, 2021, 30 respondents had undertaken satisfactory completion of the questionnaire completed the questionnaire satisfactorily with no apparent difficulty.

### Sample size calculation

To calculate a sample size for this survey, a pilot study was used. The questionnaire was sent to 50 community pharmacists from which 30 respondents were achieved, yielding a response rate of 60%. The sample size calculation was based on the question” Do you have previous exposure and experience in dealing with medication disasters?”According to the pilot study, the proportion of people who answered yes to this question was approximately 60%. The alpha level was set at 5%, giving a 95% confidence interval. Precision (D) for the 95% confidence interval was fixed at 5% so that the 95% CI would have a maximum width of 10%. Based on these assumptions, a sample size n of 527 was required, assuming that nonresponse rates would be approximately 30%.

### Sampling technique

To ensure representativeness, this study used a random sampling technique. In 2010, it was estimated that a total of 2000 community pharmacies are practicing across the UAE [17]. The contact details and locations of community pharmacies in the areas chosen for study were taken from local business directories and the Yellow Pages.

The stratification in the current study involved the division of the community pharmacies that are practicing across the UAE into groups or strata based on the community pharmacies’ locations. Accordingly, three strata were identified: community pharmacies located in Abu Dubai, community pharmacies located in Dubai, and community pharmacies located in the Northern Emirates.

Each pharmacy was given an ID number, after which all the listed pharmacies were subjected to a simple random sample selection process. Pharmacies selected for inclusion were then categorized by type and location. Once pharmacies had been selected, Excel software was used to record all related data to serve as a sampling frame, reporting the name, type, location, email address, and phone number of each pharmacy each pharmacy’s name, type, location, email address, and phone number.

### Data collection

Selected community pharmacies across, Abu Dubai, Dubai, and the Northern Emirates were visited between 28 January 2021 and 15 October 2021. The researchers explained the research purpose to the pharmacists and noted their email addresses. Face-to-face interviews were then carried out and a structured questionnaire was used.

### Research instrument development

After referring to previous similar studies [18–20]. About medicine preparedness and readiness in the literature, a structured questionnaire was designed and adapted to cover all the main key points of the research in a way that suits the local population of the UAE. Experts reviewed the final version in the field to ensure that the content relevance and design were of an acceptable standard. Furthermore, content relevance and appropriateness were approved by four faculty members from the Faculty of Medicine and Clinical Pharmacy at Ajman University. Small changes were made on the advice of the experts consulted.

The questionnaire’s content validity was also tested against Lawshe’s content validity [21], with all items reporting a content validity ratio (CVR) of 0.78. Under Lawshe’s method [21], any items scoring a CVR of ≥ 0.78 are acceptable; items not meeting this threshold are usually removed from the research instrument. A content validity index (CVI) is then calculated from the mean of all items used in the final research instrument with acceptable CVR values. The questionnaire designed for the current study had a final CVI of 0.879 and therefore passed the threshold [22]. A pilot study was then carried out to test the face validity with 10 community pharmacists whose data were excluded from the final analysis. Cronbach’s α value was calculated to analyze the research instrument’s reliability, with a score of 0.76 indicating that internal consistency was acceptable.

### Research instrument sections

The questionnaire was divided into two parts, covering

1. Seven questions to elicit demographic information, covering gender, position held in the pharmacy, number of years experience, and which university they had graduated from
2. Twenty questions 20 designed to assess the community’s knowledge, attitude, and practice about disaster medicine preparedness and readiness

### Questionnaire scoring

The knowledge about medicine preparedness and readiness was determined by asking the participants, “Do you have previous exposure and experience in dealing with disasters”. Answers to the question addressing knowledge (“yes”/“no”/“don’t know”), with all yes answers scoring “1” and No or don’t know scoring “0.”

20 items measured attitude and practice regarding medicine preparedness and readiness. These items were rated on the 5ts Likert scale (0 = “strongly disagree”, 1 = “disagree”, 2 = “neutral”, 3 = “agree”, 4 = “strongly agree”). The raw scores of 0 to 4 were calculated for each respondent by summing the grading for the 20 items. The percentage was calculated, representing a range of 0% to 100%, approximating the overall attitude and practice among community pharmacists.

The level of attitude and practice among the community pharmacists was determined by generating a median score to classify them into “Good attitude and practice” and Poor attitude and practice.” The Median score obtained was 55. Hence, respondents who scored 55 marks and above were considered to have a good attitude and practice, whereas those who scored less than 55 marks were considered poor attitude and practice. These dichotomous outcome variables mentioned above were used in logistic regression analysis models.

### Ethical considerations

The Institutional Ethical Review Committee of Al-Ain University approved the study. Before data collection, the purpose of the survey was explained, and they were informed that completion and submission of the questionnaire would be undertaken upon their consent. All participants signed the informed consent. No participant identities were recorded and confidentiality was guaranteed.

### Statistical analysis

SPSS Version 24 was used to analyze the data collected. Frequencies (stated as percentages) were used to summarize qualitative variables, whereas ± standard deviation (±SD) was used to summarize quantitative variables. To test the normality of the data we used a Shapiro-Wilk test (with a p-value less than 0.05 confirming the normality of the continuous variable) or by visual assessment of the Normal Q-Q Plot. A Chi-square test was used to determine the relationship between knowledge and demographic factors. Logistic regression models were used to determine the factors influencing the knowledge, attitude, and practice of medicine preparedness and readiness. The stepwise method was used for variable selection and model building. A p-value < 0.05 was chosen as the cut-off for statistical significance.

## Results

### Demographic characteristics

Demographic information is shown in Table 1. A total of 500 pharmacists participated in the study. This gave a response rate of 94.8% (500/527). Of the total, 39.8% (n = 199) aged 20–25 years old, 56.2% (n = 281) aged 26–30 years old and 4% (n = 20) aged ≥ 31 years old. The majority of the participant were female (71.4%) and most of them were bachelor’s degree holders (85.4%). Among the participants, 237 (47.4%) worked in Independent Pharmacies and 263 (52.6%) from chain pharmacies. The vast majority were Pharmacists (84.4%) and graduated from local universities (94.4%). The experience years amongst the study participants were detailed as follows: 61 (12.2%) had less than one year of experience, 200 (40%) had 1–5 years, 60 (12%) had 6–10 years, 126 (25.2%) had11-15 Years and 53 (10.6%) had16 or more years experience.

**Table 1:** Number and percentages of the questions on Demographic characteristics (N = 500).

Baseline characteristics	Responses	F	%
**Gender**	Male	143	28.6%
	Female	357	71.4%
**Age**	20–25 years old	199	39.8%
	26–30 years old	281	56.2%
	≥ 31 years old	20	4%
**Education Level**	Bachelor	427	85.4%
	Postgraduate	73	14.6%
**Pharmacy type**	Independent Pharmacy	237	47.4%
	Chain Pharmacy	263	52.6%
**Position**	Chief pharmacist	78	15.6%
	Pharmacist in charge	422	84.4%
**Years of experience**	Less than one year	61	12.2%
	1–5 Years	200	40%
	6–10 Years	60	12%
	11–15 Years	126	25.2%
	16 years and above	53	10.6%
**University of graduation**	Local	472	94.4%
	Regional/ International	28	5.6%

**Abbreviations:** F, frequency; %, percentage.

### Knowledge about disaster medicine preparedness and readiness

The average knowledge score was 25.6% with a 95% confidence interval (CI) of [21.7%, 29.4%]. The knowledge about disaster medicine preparedness and readiness was evaluated by asking the participants “Do you have previous exposure and experience in dealing with disasters”.

Table 2 displays the logistic regression analysis results for the factors that influence the community pharmacists’ knowledge about disaster medicine preparedness and readiness.

**Table 2:** Participants’ knowledge according to demographic variables.

Demographic	Groups		Participants knowledge			
		Estimate	OR	95% CI		P-value
				Lower	Upper	
**Gender**	Male	55 (38.5%)	2.432	1.591	3.715	< 0.001*
	Female	73 (20.4%)	**Ref.**	-----	-----	-----
**Age**	20–25 years old	43 (21.6%)	**Ref.**	-----	-----	-----
	26–30 years old	76 (27%)	1.345	0.877	2.064	0.175
	≥ 31 years old	9 (45%)	2.968	1.156	7.625	0.024*
**Educational level**	Bachelor	89 (20.8%)	**Ref.**	-----	-----	-----
	Postgraduate	39 (53.4%)	4.356	2.601	7.296	< 0.001*
**Pharmacy type**	Independent Pharmacy	101 (42.6%)	6.491	4.040	10.429	< 0.001*
	Chain Pharmacy	27 (10.3%)	**Ref.**	-----	-----	-----
**Position**	Chief pharmacist	36 (46.2%)	3.075	1.862	5.077	< 0.001*
	Pharmacist in charge	92 (21.8%)	**Ref.**	-----	-----	-----
**Years of experience**	Less than one year	13 (21.3%)	**Ref.**	-----	-----	-----
	1–5 Years	50 (25.0%)	1.231	0.616	2.457	0.556
	6–10 Years	11 (18.3%)	0.829	0.338	2.031	0.681
	11–15 Years	33 (26.2%)	1.310	0.631	2.719	0.468
	16 years and above	21 (39.6%)	2.423	1.063	5.522	0.035*
**University of graduation**	Local	110 (23.3%)	**Ref.**	-----	-----	-----
	Regional/ International	18 (64.3%)	5.924	2.657	13.209	< 0.001*

**Notes:** P-values less than 0.05 were considered statistically significant

**Abbreviations**: OR, odds ratio; CI, confidence interval.

The results of this procedure showed that better knowledge scores were observed in the male gender (OR 2.43; 95% CI 1.05–3.72), participants aged ≥ 31 years old (OR 2.97; 95% CI 1.16–7.6), postgraduates (OR 4.36; 95% CI 2.6–7.3), participants from independent Pharmacies (OR 6.5; 95% CI 4.04–10.43), chief pharmacists (OR 3.1; 95% CI 1.86–5.07), participants with 16 years and more experience years (OR 2.42; 95% CI 1.063–5.522) and participants who graduated from regional/international universities (OR 5.92; 95% CI 2.65–13.2).

### Attitude and practice about disaster medicine preparedness and readiness

The average attitude and practice score was 67% with a 95% confidence interval (CI) of [65.6%, 86.2%]. The attitude and practice toward disaster medicine preparedness and readiness among the community pharmacists were evaluated by asking the participants 20 questions.

Table 3 shows the univariate and multivariate regression analysis for the factors influencing the attitude and practice of disaster medicine preparedness. To select the set of the factors that jointly influence the attitude and practice of disaster medicine preparedness among community pharmacists, we used the stepwise procedure applied to the multivariate logistic regression model. The results of this procedure showed that better attitude and practice about disaster medicine preparedness were observed in postgraduates (OR 2.54; 95% CI 1.26–1.44), pharmacists from independent pharmacies (OR 1.35; 95% CI 2.43–2.66), and chief pharmacists (OR 1.26; 95% CI 1.17–1.35).

**Table 3:** Univariate and multivariate analysis of factors associated with attitude and practice about disaster medicine preparedness and readiness.

Factors	Attitude and Practice score (≥ 55)								
	Univariate					Multivariate			
	OR	95% CI			P-value	OR	95% CI		P-value
Gender **(Ref. Female)**									
Male	1.122	1.071		1.176	0.014	**----------**	**----------**	**----------**	**----------**
**Age** (**Ref. 20–25 years)**									
26–30 years old	1.160	1.111		1.211	0.021	**----------**	**----------**	**----------**	**----------**
≥ 31 years old	1.549	1.379		1.740	0.039	**----------**	**----------**	**----------**	**----------**
**Education (Ref. Bachelor)**									
Postgraduate	1.767	1.656		1.885	< 0.001*	1.346	1.257	1.442	0.001*
**Pharmacy type(Ref. Chain Pharmacy)**									
Independent pharmacy	2.812	2.690		2.938	< 0.001*	2.543	2.427	2.664	0.013*
**Position (Ref. Pharmacist in charge)**									
Chief pharmacist	1.955	1.834		2.085	0.001	1.258	1.173	1.348	0.017*
**Years of experience (Ref. Less than one year)**									
1–5 Years	1.066	0.997		1.139	0.048	**----------**	**----------**	**----------**	**----------**
6–10 Years	1.127	1.037		1.225	0.029	**----------**	**----------**	**----------**	**----------**
11–15 Years	1.458	1.334		1.593	0.031	**----------**	**----------**	**----------**	**----------**
16 years and above	1.458	1.334		1.593	0.005	**----------**	**----------**	**----------**	**----------**
**University of graduation (Ref. Local)**									
Regional /International	2.096	1.885	2.332		< 0.001*	1.281	1.144	1.435	< 0.001*

**Notes:** P-values less than 0.05 were considered statistically significant, “---“not included in the multivariate logistic regression model **Abbreviations**: OR, odds ratio; CI, confidence interval.

Table 4 shows the results of each question related to attitude and practice about disaster medicine preparedness.

**Table 4:** Number and percentage of the questions on attitude and practice items.

Attitude and Practice items	Strongly disagree		disagree		Neutral		agree		Strongly agree	
	F	%	F	%	F	%	F	%	F	%
1. I consider myself prepared for the management of disasters medicines	17	3.4	62	12.4	75	15.0	273	54.6	73	14.6
2. I would feel confident in my abilities as a pharmacist in disaster medicines situation	28	5.6	92	18.4	98	19.6	204	40.8	78	15.6
3. I would be interested in educational classes on disaster medicines preparedness that relates specifically to the country’s situation	33	6.6	120	24.0	151	30.2	168	33.6	28	5.6
4. I would be considered a key leadership figure in my community in a disaster medicines situation	37	7.4	119	23.8	93	18.6	202	40.4	49	9.8
5. I have personal/family emergency plans in place for disaster medicines situations	12	2.4	59	11.8	80	16.0	221	44.2	128	25.6
6. I would feel confident as a future manager or coordinator of a shelter/healthcare/ medication supply facility	29	5.8	51	10.2	85	17.0	212	42.4	123	24.6
7. I would be willing to be a future member of a healthcare facility/team in case of a medical disaster	7	1.4	46	9.2	106	21.2	228	45.6	113	22.6
8. I would feel confident implementing emergency and disaster medicine plans and procedures	20	4.0	67	13.4	103	20.6	203	40.6	107	21.4
9. would feel confident in providing medicine-related education in case of disaster or emergency	38	7.6	75	15.0	118	23.6	189	37.8	80	16.0
10. As a community pharmacist, I consider myself prepared for the management of medical disasters	6	1.2	39	7.8	75	15.0	184	36.8	196	39.2
11. As a community pharmacist, I would feel confident in my abilities as a future healthcare provider and first responder in medical disaster situations	10	2.0	26	5.2	63	12.6	149	29.8	252	50.4
12. There’s enough awareness on “ways to stand pandemics and other h and natural emergencies among healthcare systems	4	0.8	22	4.4	60	12.0	169	33.8	245	49.0
13. I need more workshops and simulated training to be ready for dealing with disaster medicines	19	3.8	61	12.2	77	15.4	181	36.2	162	32.4
14. My role in disaster medicines situations is clear	18	3.6	56	11.2	83	16.6	191	38.2	152	30.4
15. I am ready to handle whatever potential risks or emergencies exist in the community	16	3.2	38	7.6	50	10.0	182	36.4	214	42.8
16. I attended workshops/seminars about disaster medicine and it is enough for me to practice in a real situation	18	3.6	25	5.0	79	15.8	218	43.6	160	32.0
17. My undergraduate coursework enables me to be ready to practice in the settings of disaster	52	10.4	69	13.8	88	17.6	175	35.0	116	23.2
18. Other extracurricular resources (eg: internet, TV, radio, and newspapers) enable me with a sufficient degree of readiness to practice under disaster	58	11.6	74	14.8	101	20.2	185	37.0	82	16.4
19. I’m ready to practice under disaster knowing that some basic medications may not be available because of the disaster situation.	43	8.6	65	13.0	107	21.4	173	34.6	112	22.4
20. I need to be more trained in providing patient-centered care in the situation of disaster medicines	39	7.8	71	14.2	120	24.0	176	35.2	94	18.8

**Abbreviations:** F, frequency; %, Percentage.

## Discussion

Irrefutably, the reduction of disaster risk is a critical component of socioeconomic growth, mainly to guarantee the future sustainability of development. Pharmacists are at the forefront of disaster patient care; hence this study was performed to evaluate the knowledge, attitude, and practices of a representative sample of community pharmacists towards disaster medicine preparedness and readiness across the UAE. Medical involvement in disaster management aims to minimize, or avoid, the potential life losses from different kinds of disasters and provide adequate medical support to victims of disaster, and achieve rapid and effective recovery where possible. Owing to threats and the multifaceted nature of the disaster, hospitals and other healthcare organizations must be equipped and organized to care for those in need of medical services and protect individuals from being exposed to any further risk [23]. Countries—LMICs particularly—must enhance their efforts to train healthcare professionals for disaster management and reduction. Community pharmacists are essential members of health care teams, especially during disasters. According to the American Society of Health-System Pharmacists, pharmacists should develop guidelines for diagnosing and treating casualties [24]. They should help in selecting appropriate medications and related supplies in emergency-preparedness programs. Moreover, ensuring proper managing and dispensing of emergency supplies of all medication.

The majority of the participants in this study were female (71.4%), and most of them were bachelor’s degree holders, aged 26–30 years old, work in chain pharmacies, and have 1–5 Years of experience. Overall, the vast majority of the respondents hold the pharmacist position in charge (84.4%) and graduated from local UAE universities. These demographic variables are consistent with those reported in studies on community pharmacists’ knowledge, attitude, and practices regarding COVID-19 pandemic management. The proportion of female pharmacists in this study was high, which is consistent with results from the studies of Vietnam (78.2%) [25], Jordan (78%) [26], Goa (79.5%) [27], Cairo (70%) [28] and Lebanon (85.2%) [29]. The fact that the number of females is exceedingly surpassed the males is characteristic of the pharmaceutical industry [25].

In addition, this study showed that male gender, participants aged ≥31 years old, postgraduates, participants from independent pharmacies, chief pharmacists, participants with 16 years and more experience years and participants who graduated from regional/international universities significantly influenced the knowledge of the community pharmacists about disaster medicine preparedness and readiness. This implies that experience and education level significantly affects the knowledge score of the respondents in this study. The important rationale behind this is the scholarly level of education and years involved in opening outlets, selling medications, and resolving health issues in the community [25]. This contravenes several studies that reported education level was not significantly associated with the knowledge score of pharmacists in several countries like the United Arab Emirates [30], Pakistan [31], and Saudi Arabia [32].

Healthcare institutions and personnel must be primed for the challenges associated with disaster management [23]. They are obligated to have the requisite knowledge, positive attitude, and practice towards disaster to be ready and prepared. The community expects the healthcare staff to be on hand to offer care for them all through the catastrophe. Thus, their capacity and readiness to effectively function are critical. Hence, this study is the first cross-sectional study to measure knowledge, attitude, and practice toward disaster medicine preparedness and readiness among community pharmacists in the UAE. Knowledge of community pharmacists regarding disaster medicine preparedness and readiness is extremely important. Several studies about disaster medicine preparedness and readiness among health care workers [33–36]. However, there was no previous study about this issue among community pharmacists in the UAE.

The majority of current research on disaster medicine highlights the COVID-19 pandemic as a prime example. For instance, Alnajjar et al. reported an appropriate average knowledge and practice toward COVID-19 among the community and hospital pharmacists in the UAE [37]. The study revealed that 45.7% of the participated pharmacists expected to have a good level of knowledge about COVID-19 transmission, symptoms, and treatment. Pharmacists aged ≥40 years old with experience of ≥10 years in the pharmacy field were found to be more knowledgeable as regards COVID-19 with higher scores of p <0.001 and p = 0.001, respectively. In Lebanon, Zeenny et al. earlier reported an appropriate level of knowledge and good practice towards COVID-19, among the respondents from Lebanese hospitals. Most of the respondents were concerned about getting infected along with their families due to their professional exposure [29]. On the contrary, the majority of community pharmacists evaluated by Muhammad et al., in a cross-sectional survey in 2 provinces of Pakistan had good knowledge but had a poor attitude and practice toward the management of COVID-19 [31]. Rayes and Abduelkarem found that collaboration between community pharmacists and physicians in Dubai enhances patients’ drug therapy outcomes [38]. Nguyen et al., surveyed the knowledge, attitude, and practices of Vietnamese pharmacists regarding the COVID-19 pandemic, and found that the pharmacists’ knowledge of COVID-19 transmission, symptoms, and prevention was good [25]. These earlier contemporary studies posit similar deductions to this current study in terms of preparedness, knowledge, attitude, and practice of disaster medicine. Nonetheless, a country like Pakistan, which is among the most vulnerable countries in South Asia due to its exposure to different kinds of disasters, is expected to be more knowledgeable and prepared for disaster management and control as compared to UAE.

Although a similar study [39] showed that 78.2% of the respondents (i.e. community pharmacists) acknowledge that the UAE is prone to a disaster which is in line with an earlier report [40] from Yemen were 85.5% of the respondents responded the same, this study found that only 25.6% of the community pharmacists knew disaster medicine preparedness and readiness. This average score of 25.6% is low in knowledge and contradicts earlier reports. Specifically, the results revealed better knowledge scores in the male gender were observed. The average attitude and practice score was observed (67%). The results further indicate that better attitudes and practices about disaster medicine preparedness were observed in postgraduates, pharmacists from independent pharmacies, and chief pharmacists. In addition, this study reported that 69.2% consider themselves prepared for the management of disaster medicines, 56.4 are confident in their pharmaceutical abilities in disaster situations, and 76% were prepared for the management of medicines disasters. This conforms to the study by [37] Gillani et al., which that reported 45.1% of the respondents to know who to contact in case of disaster, and is considerably higher than the 35.5% documented by Al-Ali &Abu Ibaid for Jordan [41]. This result is imperative as WHO emphasized that hospitals, other healthcare institutions, and personnel must have the knowledge, positive attitude, and readiness to practice towards disaster [42].

In general, the responses to questions on attitude and practice items indicate that the respondents’ level of readiness and preparedness was moderate to high. Similarly, related studies have demonstrated a moderate readiness to practice among the healthcare staff [43–45]. Consequently, it can be strongly inferred that improving the education level, position, and years of experience of community pharmacists may strengthen their knowledge, attitude, and practice of disaster medicine. The study had some limitations that should be highlighted. First, a cross-sectional survey approach may not generate sufficient data to reach a strong conclusion. Therefore, additional longitudinal research studies are recommended. Second, the use of a survey instrument that contained closed-ended questions may have resulted in the omission of certain critical considerations. Nonetheless, to the best of the author’s knowledge, this study represents the first of its kind to examine pharmacists’ perspectives on disaster medicine readiness in the United Arab Emirates, and it provides useful insights that will be informative for health policymakers and planners.

## Conclusions

To conclude, the occurrence of disasters cannot be predicted or adequately managed without preparedness. Hence, pharmacists and all other health care workers should exercise their responsibilities in preparing for and responding to disasters and all other unusual circumstances. Moreover, it’s essential to provide a continuing education program using different educational strategies improved community pharmacy competencies (e.g. knowledge, attitudes, and perceptions) to improve the skills and practices regarding disaster medicine preparedness and readiness.

## Supporting information

S1 Data (XLSX)
