# Correction: Exploring the knowledge, attitude and practice towards disaster medicine preparedness and readiness: A prescriptive insight by the community pharmacists in the United Arab Emirates

**DOI:** 10.1371/journal.pone.0315214

**Published:** 2024-12-05

**Authors:** Ammar Abdulrahman Jairoun, Sabaa Saleh Al-Hemyari, Moyad Shahwan, Nasser M. Alorfi, Faris El-Dahiyat, Md. Sanower Hossain, Miamona Jairoun, Ammar Ali Saleh Jaber

The fourth author’s name is spelled incorrectly. The correct name is: Nasser M. Alorfi. The correct citation is: Jairoun AA, Al-Hemyari SS, Shahwan M, Alorfi NM, El-Dahiyat F, Hossain MS, et al. (2022) Exploring the knowledge, attitude and practice towards disaster medicine preparedness and readiness: A prescriptive insight by the community pharmacists in the United Arab Emirates. PLOS ONE 17(8): e0273209. https://doi.org/10.1371/journal.pone.0273209
